# The effect of peat replacement in horticulture media by willow (*Salix viminalis* L.) biomass compost for cucumber transplant production

**DOI:** 10.3389/fpls.2024.1348073

**Published:** 2024-02-12

**Authors:** Katarzyna Adamczewska-Sowińska, Józef Sowiński, Elżbieta Jamroz, Jakub Bekier

**Affiliations:** ^1^ Department of Horticulture, Wroclaw University of Environmental and Life Sciences, Wrocław, Poland; ^2^ Institute of Agroecology and Plant Production, Wroclaw University of Environmental and Life Sciences, Wroclaw, Poland; ^3^ Institute of Soil Sciences Plant Nutrition and Environmental Protection, Wroclaw University of Environmental and Life Sciences, Wrocław, Poland

**Keywords:** alternative horticulture media, mixture proportion, cucumber, transplant parameters, willow

## Abstract

This research evaluated the usefulness of horticultural substrates prepared on the basis of compost from chipped willow without additives and with the addition of nitrogen and decomposing mycelium of the cellulose-lignin fraction of wood in the cultivation of cucumber seedlings. The produced composts were mixed in different proportions: mixture 1 (W1) - the proportion of compost without additives and compost prepared with the addition of nitrogen and mycelium was in the ratio of 50:50, mixture 2 (W2) - the proportion of compost without additives and compost prepared with the addition of nitrogen and mycelium was in the ratio of 75:25. The starting mixtures were used to prepare horticultural substrates with different components (peat - P, vermicompost - V) and additives: basaltmeal - B, biochar from deciduous wood - C. The components were added in varying proportions. A total of 29 different substrates were subsequently tested in the study. Plant showed that the traits assessed varied to a greater extent under the effect of the test factors than at earlier growth stages. It was demonstrated that cucumber grown on substrates with 75% or 50% willow compost had a unit weight at the same statistical level as when grown on peat substrate (P). The plants with the highest unit weight (8.5- 10.4 g), belonged to the same homogeneous group and derived from sites W1P1B2, W2P1, W1P1B1, W2P2, W1P1C1, P, W1P1, W2B1, W2P2B2. High-quality cucumber transplant should characterise well develop, optimal height-to-stem thickness ratio, short hypocotyl, thick green leaves and cotyledons.

## Introduction

Peat, a widely used substrate component in horticulture, has faced criticism due to environmental concerns and ongoing climate change (Hong and Gruda, 2020; Gruda, 2021). For example over the past decade (2011-2021), the proportion of peat in total horticultural media in the UK has decreased by 26 percentage points (pp) and currently accounts for 36% of the total (Holmes and Bain, 2023). Therefore, the quest for peat-based substrate additives or alternatives to peat for new horticultural applications has intensified. In this regard, research is underway on environmentally safe composts, biochar, agricultural waste compost, and vermicompost (Gruda et al., 2009).

Earlier studies have shown the suitability of cellulose-lignin biomass composts from willow (*Salix viminalis* L.) for the production of substrates used in tomato and cucumber transplant cultivation (Adamczewska-Sowińska et al., 2021; Adamczewska-Sowińska et al., 2022). The carbon content of willow biomass ranges from 502-530 g per kg dry weight while the nitrogen content ranges from 3.9 to 7.5 g per kg dry weight (Krzyżaniak et al., 2015; Liu et al., 2016; Weger et al., 2016; Larsen et al., 2019; Stolarski et al., 2020a). The broad carbon:nitrogen ratio in biomass of 125:1 based on data presented by Stolarski et al. (2020b) needs to be corrected in order to start the biomass composting process properly. An appropriate C:N ratio is a very important parameter influencing the growth of microorganisms and the optimum ratio for their development is considered to be in the range of 25-35. One that is too high (above 35:1) may lengthen the biotransformation period and one that is too low (below 20) increases nitrogen losses due to faster mineralisation of biomass (Bernal et al., 2009; Osono, 2015; Arnstadt et al., 2016; Akratos et al., 2017).

By using alternative organic materials in horticultural substrates (such as compost, wood or plant fibre), microbial biomass may increase and contribute to reduced compost stability (Agarwal et al., 2021; Vandecasteele et al., 2022). In addition, preliminary sanitation treatments are needed to ensure the destruction of weed seeds, pathogens. It is recommended to carry out treatments such as acidification or thermal phase during composting before preparing the substrates (Grunert et al., 2016; Vandecasteele et al., 2018). During the composting process, increasing the temperature to 35-40°C also promotes microbial biodiversity (Storey et al., 2015), while further temperature elevation to 45-55°C enhances the rate of biomass biodegradation and accelerates mineralisation (Ryckeboer et al., 2003). The addition of mineral nitrogen provides optimum conditions for composting of willow biomass (Sowiński et al., 2022). Too high a nitrogen content can cause salinisation of the substrate and have a negative effect on the growth of tomato plant transplants (Adamczewska-Sowińska et al., 2021).

The use of composts as a major substitute for peat or coconut fibre and the provision of optimum parameters is an essential requirement to be met in the preparation of a good horticultural growing medium (Vandecasteele et al., 2021). The optimum pH, salinity and nutrient content are vital. Meeting these parameters requires the selection and blending of suitable composts or a variety of both organic and mineral materials (Vandecasteele et al., 2021). In a study by Giménez et al. (2020), replacing peat as a growing medium component in leafy vegetable production with 25% compost had a beneficial effect on yield and crop quality. Similarly, Tüzel et al. (2020) found that compost from olive production waste could replace peat in the mixture as a substrate for tomato seedling production in 25%. Replacing peat with vermicompost or biochar did not have a negative effect on horticultural plant growth (Alvarez et al., 2017).

The primary goal of the research was to obtain a new substrate that would allow the production of cucumber seedlings with quality parameters similar to those in peat substrate. The aim of the study was to find answers to the question of the quality of cucumber seedlings produced in substrates prepared based on composts from willow biomass, combined in various proportions with peat or vermiculite, as well as with additions of biochar or basalt meal.

On the basis of the formulated objective, the research hypotheses were formulated: a mixture of willow composts will provide similar conditions for the growth of cucumber seedlings as peat substrate. The addition of milled basalt rock, vermicompost and biochar will improve the parameters of cucumber seedlings and significantly reduce the use of peat in seedling production and may improve growing conditions.

## Methodology

In 2019, four willow chip-based composts were produced: compost from willow chips alone and compost with the addition of nitrogen and the wood-decomposing mycelium *Peniophora gigantea* of the Mycelium and Biopreparations Factory ‘POSZWALD’. The favourable effect on the composting process by the additives used was due to the broad C:N ratio of 118:1 in the willow biomass (Adamczewska-Sowińska et al., 2021).

Two mixtures with different proportions of compost (volume ratios) were prepared (**Table 1**):

**Table 1:** Proportion of individual components in the tested media.

Code	Horticulture media proportion (% of volume capacity)			Additives (g per 100 g of horticulture media)	
	willow compost	peat	Vermicompost	basaltmeal	biochar
Media component and their percentage					
P	–	100	–	–	–
V	–	–	100	–	–
W1	100	–	–	–	–
W2	100	–	–	–	–
W1P1	50	50	–	–	–
W1P2V2	50	25	25	–	–
W1V1	50	–	50	–	–
W2P1	50	50	–	–	–
W2P2V2	50	25	25	–	–
W2V1	50	–	50	–	–
W2P2	75	25	–	–	–
W2P3V3	75	10	15	–	–
W2V2	75	–	25	–	–
Media with additives					
W1B1	100	–	–	5	–
W1B2	100	–	–	10	–
W1P1B1	50	50	–	5	–
W1P1B2	50	50	–	10	–
W2B1	100	–	–	5	–
W2B2	100	–	–	10	–
W2P2B1	75	25	–	5	–
W2P2B2	75	25	–	10	–
W1C1	100	–	–	–	10
W1C2	100	–	–	–	20
W1P1C1	50	50	–	–	10
W1P1C2	50	50	–	–	20
W2C1	100	–	–	–	10
W2C2	100	–	–	–	20
W2P2C1	75	25	–	–	10
W2P2C2	75	25	–	–	20

- in mixture 1 (hereafter referred to as W1), the proportion of compost without additives and compost prepared with nitrogen and mycelium was 50:50.
- in mixture 2 (hereinafter referred to as W2) - the proportion of compost without additives and compost prepared with nitrogen and mycelium was 75:25.

Horticultural substrates for growing cucumber seedlings were prepared using mixtures W1 or W2 with different additives. These were.

- peat (P), the proportion of which varied and was 50, 25 and 10% in volume percentage, referred to as P1, P2, P3 respectively.
- vermicompost (V), the proportion of which varied and in volume percentages was 50, 25 and 15%, labelled V1, V2, V3 respectively.

Two groups were separated for substrate preparation and mineral additives of milled basalt rock and biochar were applied to one.

- milled basalt rock meal (basaltmeal) (B) at 5 g per 100 g of substrate - B1, and 10 g per 100 g of substrate - B2.
- biochar prepared from waste deciduous wood (C) at 10 g per 100 g of substrate - C1, and 20 g per 100 g of substrate - C2.

In 2021, experimentation on the obtained media commenced. On 7 April, cucumber seeds of the Hermes Skierniewicki F1 cultivar were spot sown into pots filled with the prepared growing media. Each variant with horticultural media was repeated 10 times.

Biometric measurements of cucumber plants were conducted on the dates April 20, April 27, and May 4, respectively, 13, 20, and 27 days after the seed sowing date. On 10 plants from each combination, height and lateral extent were assessed using a measuring tool with an accuracy of 0.1 cm, along with the leaf count. Based on these measurements, the growth increment of each parameter between the mentioned measurement dates was calculated, contributing to the characterization of the plant growth rate. In the last measurement session, before terminating the experiment, the stem diameter was also evaluated at a height of 0.5 cm from the plant base, and the individual plant mass was determined. The ratio of plant height to stem diameter was calculated – an index describing the condition of the plants. Electronic calipers and a precision electronic scale (accuracy 0.1 g) were used for these measurements.

### Statistical analysis

Data from the morphological measurements of plants and the quantitative and qualitative evaluation of seedlings collected at individual dates and before harvest were subjected to ANOVA/MANOVA analysis in Statistica software (version 13.1, StatSoft, Poland). All analyses were performed at a significance level of p < 0.05. 1- and 2-way analysis of variance was performed to assess the effect of medium type and different proportions of components in the medium mixture on the determined cucumber seedling parameters. The substrates and proportions in the mixture corresponded to the fixed effect of the model, while the repetitions accounted for the variable effect of the model. The analysis assessed the effect of the proportion of willow compost, peat, vermicompost, basaltmeal and biochar as a substrate component as compared to homogeneous media.

## Results

Thirteen days after sowing the seeds, the majority of cucumber seedlings across the different sites exhibited a similar stage of development, characterized by well-developed and distributed cotyledons and the appearance of the first true leaf. Between 90 - 100% of the seedlings had the first true leaf: W2P2, W2V2, W2B1, W2P2B2, W1P1C1, W2C2 and W2P2C2, and 88 - 89% at sites W2C1 and W1C1 (**Tables 2** and **2A**). Conversely, the fewest plants with the first leaf were recorded in sites: W1, W1P2V2 (33 - 44%), W2B2, W2P2B1 (50%) and V and P (56%).

**Table 2:** The effect of horticultural media on cucumber transplant at 13 Days After Sowing (DAS).

Code	Lateral extent (cm)	Height (cm)	Average leaves number
P	6.9abcde*	3.4bcd	0.6
V	6.1ab	4.0de	0.6
W1	5.8a	3.3bcd	0.3
W2	7.3bcde	3.7cde	0.8
W1P1	8.1de	3.7cde	0.7
W2P1	6.7abc	2.8ab	0.7
W2P2	8.0cde	4.3e	0.9
W1V1	6.8abcde	2.9abc	0.8
W2V1	7.1abcde	2.4a	0.8
W2V2	8.2e	3.3bcd	0.9
W1P2V2	6.7abc	3.3bcd	0.4
W2P2V2	6.5ab	2.8ab	0.6
W2P3V3	7.1abcde	4.4e	0.8
**Significance**	**P<0.05**	**P<0.001**	**n.s.**

*Means in the same column that include a lowercase letter are not statistically significant.

n.s., no significant.

**Table 2A:** The effect of horticultural media additives on cucumber transplant quality at 13 DAS.

Code	Lateral extent (cm)	Height (cm)	Average leaves number
P	6.9cdef*	3.4abcd	0.6abc
W1	5.8abc	3.3abc	0.3a
W2	7.3defg	3.7abcde	0.8bcd
W1B1	7.0cdefg	3.2abc	0.8bcd
W2B1	8.2g	4.0bcde	0.9cd
W1B2	7.0cdefg	3.4abcd	0.6abc
W2B2	6.7bcde	3.4abcd	0.5ab
W1P1B1	8.0fg	4.5e	0.8bcd
W1P1B2	7.0cdefg	4.1cde	0.6abc
W2P2B1	6.1abcd	4.1cde	0.5ab
W2P2B2	7.8efg	4.2de	1.0d
W1C1	6.9cdef	3.0a	0.9cd
W2C1	7.0cdefg	4.0bcde	1.0d
W1C2	5.4a	3.4abcd	0.6abc
W2C2	6.6abcd	4.1cde	1.0d
W1P1C1	8.0fg	3.4abcd	1.0d
W1P1C2	5.5ab	3.7abcde	0.8bcd
W2P2C1	6.6abcd	4.0bcde	0.9cd
W2P2C2	7.0cdefg	3.4abcd	0.9cd
**Significance**	**P<0.001**	**P<0.05**	**P<0.01**

*Means in the same column that include a lowercase letter are not statistically significant.

n.s., no significant.

As shown in **Tables 2** and **2A**, at the beginning of growth, the type of growing medium had no significant effect on the number of leaves in cucumber plants. It ranged from 0.3 - 0.4 leaves per plant at sites W1 and W1P2V2 to 0.8 leaves per plant (W2, W2V1, W2P3V3, W1V1) and 0.9 pcs. (W2P2, W2V2). The lateral extent of the plants was observed to vary significantly, ranging from 5.8-6.5 cm (W1, V, W2P2V2) to 8.0-8.2 cm (W2V2, W1P1, W2P2). There was, however, a noticeable tendency for plants growing in peat medium or in media with peat as a component compared to those growing in basal W1 and W2 to have a greater spread. Cucumber plants grown in W2 substrate had significantly greater height (by 25.9%) and 2.7 times more leaves compared to W1.

Notably, the greatest heights were those of plants (4.4 cm) from sites W2P3V3 and W2P2 (4.3 cm). In the same homogeneous group was the height of plants from V (4.0 cm), W2 (3.7 cm) and W1P1 (3.7 cm). In contrast, the addition of more vermicompost (V1) to the compost of W1 and W2 resulted in plants in a group with significantly lower height.

The addition of basaltmeal (B1 or B2) or peat together with a higher dose of basaltmeal (P1B2) to the W1 growing medium increased plant lateral extent by an average of 20.7%, but this difference was not confirmed statistically (**Table 2A**). However, cultivation on W1P1B1 substrate resulted in cucumber plants with the largest lateral extent (8 cm) and being among the tallest with the largest number of leaves (average 0.8). Plants grown on W2B1 and W2P2B2 substrate were also exhibited substantial growth. The addition of peat or basaltmeal to the W2 substrate did not result in significant changes in the measured cucumber plant traits.

The observations revealed that the addition of biochar to the W1 substrate at a lower dose (C1) led to a notable trend towards a 20% increase in plant extent, although this trend was not statistically confirmed. However, the incorporation of peat (P1C1) resulted in a significant increase of 37.9% in plant lateral extent. No changes were observed in plant height, while the number of leaves on average increased by more than three times. In contrast, the addition of biochar to the homogeneous W2 substrate or to its mixture with peat did not produce any significant changes in the measured cucumber plant traits.

On the second observation date, which was at 20 DAS, it was observed that the type of substrate did not have a significant effect on the lateral extension of the cucumber plants or their foliage, as shown in **Table 3**. The lateral extension of the plants ranged from 8.9 cm for W2V1 to 12.8 cm for W1P1. However, there was a noticeable tendency for plants grown in peat substrate (P) or substrates containing peat (W1P1, W2P1, W2P2) to exhibit increased lateral extent compared to those grown in basic substrates W1 and W2. In addition, it was found that plants in these sites were on average 13.8% taller. The use of vermicompost as a homogeneous substrate or substrate component did not enhance cucumber growth, with the exception of the response to W2P3V3 substrate, which contained the lowest amount of vermicompost. The plants grown in this substrate belonged to the group with the greatest height (10.2 cm) and were distinguished by their spread (11.2 cm) and number of leaves (average 1.7).

**Table 3:** The effect of horticultural media on cucumber transplant quality at 20 DAS.

Code	Lateral extent (cm)	Height (cm)	Average leaves number
P	12.0	8.2abc*	1.6
V	9.8	8.8abcd	1.3
W1	9.2	8.0abc	1.6
W2	9.6	8.7abcd	1.8
W1P1	12.8	8.9bcde	1.8
W2P1	10.3	9.2cde	1.7
W2P2	11.0	10.4e	1.6
W1V1	9.8	8.3abc	1.7
W2V1	8.9	8.4abc	1.5
W2V2	11.2	9.1bcde	1.6
W1P2V2	9.8	7.6ab	1.6
W2P2V2	10.3	7.1a	1.6
W2P3V3	11.2	10.2de	1.7
**Significance**	**n.s.**	**P<0.01**	**n.s.**

*Means in the same column that include a lowercase letter are not statistically significant.

n.s., no significant.

The statistical analysis of the experimental data confirmed that the addition of basaltmeal or biochar to the W1 or W2 substrate mixed with peat had a significant effect on the cucumber plant quality (refer to **Table 3A**). In the WPB media treatment, the lateral extent of the plants increased by an average of 20.9% compared to the plants grown in WB, while in WPC, the lateral extent increased by 11.2% compared to those in WC. Moreover, the foliage of WPB plants was on average 12.3% larger than in WB.

**Table 3A:** The effect of horticultural media additives on cucumber transplant quality at 20 DAS.

Code	Lateral extent (cm)	Height (cm)	Average leaves number
P	12.0e*	8.2abcde	1.6abcd
W1	9.2abc	8.0abcd	1.6abcd
W2	9.6abcd	8.7bcdef	1.8d
W1B1	8.5a	8.1abcd	1.4a
W2B1	11.7de	11.2g	1.5ab
W1B2	8.4a	7.8abc	1.4a
W2B2	8.8ab	8.8bcdef	1.4a
W1P1B1	12.2e	11.7g	1.7cd
W1P1B2	11.1bcde	11.5g	1.6abcd
W2P2B1	10.1abcde	8.9cdef	1.4a
W2P2B2	11.6de	9.2def	1.7cd
W1C1	9.2abc	7.7abc	1.4a
W2C1	9.6abcd	9.6ef	1.4a
W1C2	8.2a	6.8a	1.5ab
W2C2	8.8ab	8.8bcdef	1.4a
W1P1C1	11.5cde	9.6ef	1.6abcd
W1P1C2	8.1a	6.8a	1.4a
W2P2C1	10.2abcde	8.2abcde	1.4a
W2P2C2	10.0abcde	7.5ab	1.5ab
**Significance**	**P<0.001**	**P<0.001**	**P<0.05**

*Means in the same column that include a lowercase letter are not statistically significant.

n.s., no significant.

The best-performing plants in terms of spread and number of leaves were obtained from sites W1P1B1, W1P1B2, W2P2B2 and W1P1C1. It was also observed that a similar lateral extent was determined in plants grown in W2B1, W2P2B1, W2P2C1, and W2P2C2 media. Furthermore, plants from media W2B1 (11.2 cm), W1P1B1 (11.7 cm), and W1P1B2 (11.5 cm) had a significantly greater height than those in other sites, with an average increase of 38.4%.

The biometric measurements conducted prior to the termination of the experiment revealed that the lateral extent and height of plants were significantly influenced by the substrate type (**Table 4**). Conversely, the number of leaves per plant, which ranged from 2.5 to 3.2, did not differ significantly among the treatments. The W2P2 substrate, consisting of 75% willow compost (W2 mixture) and 25% peat, resulted in the most vigorous seedlings, exhibiting the greatest lateral spread (27.3 cm) and height (15.5 cm). Plants grown in peat substrate and in W2P3V3 and W2V2 substrates showed similar lateral spread at a significant level, whereas height was found to be significant only in the W2P3V3 treatment. Furthermore, a trend towards reduced plant biomass was observed with an increase in the proportion of vermicompost in the substrate. Specifically, when vermicompost constituted 50% of the mixture with willow compost, the average lateral spread and height of plants were 20.4% and 9.2% lower, respectively, compared to the 25% vermicompost mixture. Similarly, when vermicompost accounted for 25% of the mixture with willow and peat, the average lateral spread and height of plants were 11% and 16.5% lower, respectively, compared to the 15% vermicompost mixture.

**Table 4:** The effect of horticultural media on cucumber transplant quality at 27 DAS.

Code	Lateral extent (cm)	Height (cm)	Average leaves number
P	27.0de*	11.3ab	3.0
V	23.1bc	12.3abc	2.7
W1	21.1abc	11.4ab	2.7
W2	23.0bc	11.3ab	3.0
W1P1	23.1bc	12.7bc	2.9
W2P1	23.4bcd	13.0cd	2.8
W2P2	27.3e	15.5e	3.0
W1V1	18.1a	10.9a	2.6
W2V1	20.6ab	11.8abc	2.5
W2V2	24.3bcde	12.5bc	3.2
W1P2V2	21.6abc	12.1abc	2.8
W2P2V2	22.4bc	11.6abc	2.9
W2P3V3	24.7cde	14.2de	2.9
**Significance**	**P<0.001**	**P<0.001**	**n.s.**

*Means in the same column that include a lowercase letter are not statistically significant.

n.s., no significant.

Cucumber plant responses to the addition of basaltmeal to the tested substrates were analysed, and the results are presented in **Table 4A**. The research revealed that the addition of basaltmeal to the W1 substrate did not lead to improvements in basic quality characteristics when present in either lower or higher doses. However, it was found that the addition of basaltmeal to the W1 mixture with peat had a positive effect on the seedlings, resulting in greater lateral extent by an average of 26.3% and increased plant height by 30% on average. Conversely, no significant variation in seedling characteristics was observed when W2 was the basic component of the growing medium. The best-quality seedlings were obtained on W1P1B1, W2P2B2, and W2B1 growing media. These plants exhibited lateral extent similar to those grown in peat substrate and, on average, 23.9% greater height and 10% more leaves.

**Table 4A:** The effect of horticultural media additives on cucumber transplant quality at 27 DAS.

Code	Lateral extent (cm)	Height (cm)	Average leaves number
P	27.0gh*	11.3bcd	3.0bcde
W1	21.1bcd	11.4bcde	2.7abc
W2	23.0def	11.3bcd	3.0bcde
W1B1	19.6abc	11.8cde	2.8abc
W2B1	25.4efgh	13.4fg	3.3def
W1B2	17.3a	10.5ab	2.6ab
W2B2	22.6cde	10.7abc	2.8abc
W1P1B1	28.4h	15.2h	3.1cdef
W1P1B2	24.9efg	14.2gh	2.9abcd
W2P2B1	22.4cde	11.7bcde	2.8abc
W2P2B2	25.3efgh	13.4fg	3.5f
W1C1	22.9def	10.4ab	2.8abc
W2C1	24.1defg	12.5def	2.8abc
W1C2	19.1ab	9.7a	2.5a
W2C2	21.5bcd	11.7bcde	2.8abc
W1P1C1	27.2gh	12.6ef	3.3def
W1P1C2	23.1def	10.9abc	2.7abc
W2P2C1	25.8fgh	11.8cde	3.0bcde
W2P2C2	24.3defg	11.0bc	3.1cdef
**Significance**	**P<0.001**	**P<0.001**	**P<0.001**

*Means in the same column that include a lowercase letter are not statistically significant.

n.s., no significant.

The results showed that the addition of biochar did not result in improvements in seedling quality. In the W1C2 media, the plant lateral extent and height, as well as the number of leaves, were the lowest among plants grown on substrates containing biochar. However, it was observed that the addition of biochar to substrates consisting of willow compost and peat promoted plant growth, especially when the addition of biochar was lower. The cucumber seedlings growth achieved on W1P1C1 and W2P2C1 substrates, matched the quality of plants grown in peat substrate. Moreover, these substrates were characterised by greater height and slightly better foliage at the W1P1C1 media.

**Figure 1:**
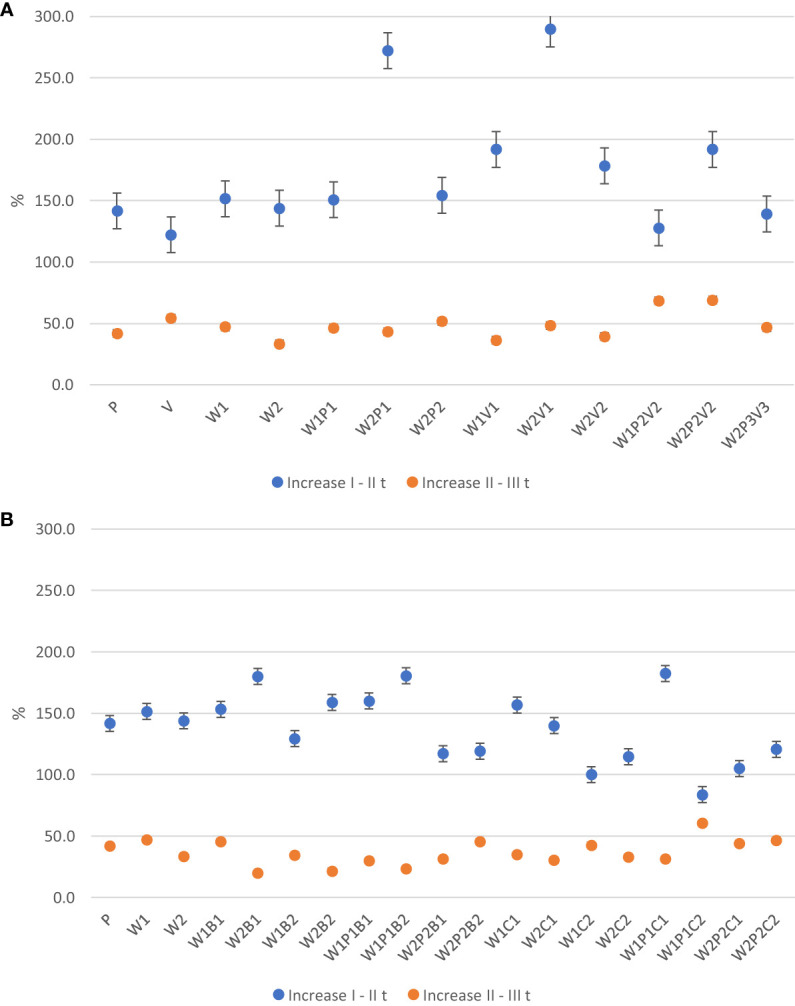
**(A)** The effect of horticultural media on cucumber plant height growth rate between measurement date. **(B)** The effect of horticultural media and additives on cucumber plant height growth rate between measurement date.

**Figure 2:**
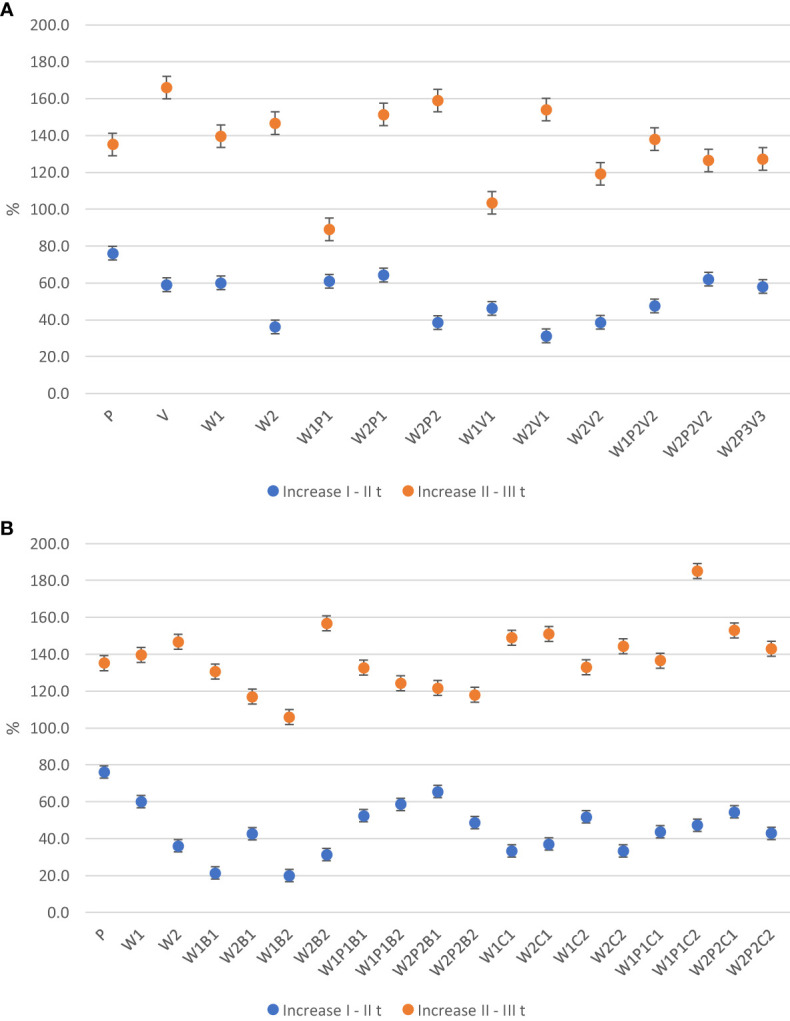
**(A)** The effect of horticultural media on cucumber plant lateral extent rate between measurement date. **(B)** The effect of horticultural media on cucumber plant lateral extent rate between measurement date.

The analysis of the experimental results revealed that cucumber plants, in their third week of vegetation, exhibited a faster rate of vertical growth compared to lateral growth, irrespective of the substrate type or additive used (see **Figures 1A, B** and **2A, B**). However, in the fourth week, a relatively smaller increase in plant height compared to lateral extent was observed. Between the first and second measurement dates, plants grown on W2P1 and W2V1 substrates, as well as those grown on W1V1 and W2P2V2 substrates, exhibited a rapid increase in plant height. Between the second and third measurement dates, the plants grown on W2P2V2 and W1P2V2 substrates exhibited the most rapid increase in plant height. Plants from W2P1 and W2V1 media showed a six-fold faster increase in height in the third week than in the fourth week, while those from W2P2V2 and W1P2V2 media grew only 1.9-2.8 times faster.

The results of the experiment indicate that in the fourth week of vegetation, the plants from sites V, W2P2, W2V1, and W2P1 demonstrated the highest lateral extent. However, the rate of lateral extent growth compared to week 3 was significantly higher on the W2V1, W2P2, and W2 substrates (4.9 - 4.1 times faster) compared to the plants from the other sites (1.5 - 3.1 times faster).

On completion of the third week of cucumber vegetation, it was observed that the addition of 10% (w/w) milled basalt to the W1 medium had a retarding effect on the growth of plant height compared to 5% (w/w). Conversely, the incorporation of the same quantity of basaltmeal into the W1 mixture with peat resulted in an acceleration of plant growth, with an increase in plant height of 180.5%. This increase was one of the highest observed. Additionally, a similar increase in plant height was observed in those cultivated on the W2 substrate with the addition of a 5% dose of basaltmeal.

The incorporation of 20% (w/w) biochar, into the W1 or W2 compost substrate and the W1P1 and W2P2 mixture led to a decrease in the rate of seedling vertical growth. However, the W1P1C1 site was an exception, where the height gain between I and II measurement was 182.4%. Between the second and third measurement dates, the height increase of cucumber seedlings was observed to be 1.4-2.6 times slower in W1P1C2, W1C2, W2P1C1, and W2P1C2 and 7.4-9.2 times slower in W2B2, W1P1B2, and W2B1 than the first and second measurement periods. The largest height increase of 60.3% was recorded in plants growing in W1P1C2.

After a three-week growth period of cucumber plants, no significant effect was observed on the rate of lateral reach growth upon the addition of basaltmeal to W1. Nevertheless, it was noted that the presence of a lower dose of basaltmeal at W2B1 and, especially, at W2P2B1 resulted in a faster increase in plant spread. At the last measurement date, only seedlings from W2B2 had a faster lateral growth rate than plants on the W2 basic substrate.

The addition of biochar to the W1 substrate significantly enhanced the lateral spread of the seedlings by the end of the production period compare o previous measurement term at W1C1 (148.9%) and W1P1C2 (185.2%) media. Transplants cultivated at W1B1, W1B2, and W2B2 exhibited distinctively faster lateral growth gains between three-week-old and four-week-old seedlings, with a 5-6.1-fold increase compared to W2P2B1 (a 1.9 fold).

**Figure 3:**
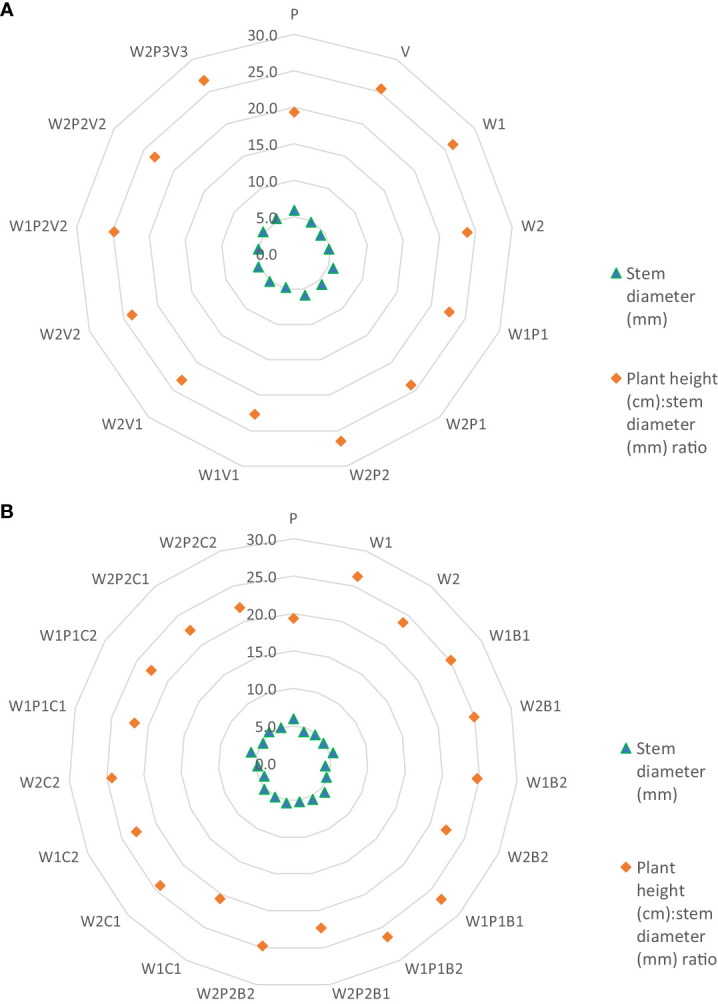
**(A)** The effect of horticulture media on stem diameter and plant height (cm):stem diameter (mm) ratio. **(B)** The effect of horticulture media and additives on stem diameter and plant height (cm):stem diameter (mm) ratio.

Upon evaluating the effect of substrate type on shoot diameter, it was determined that cucumber seedlings grown on peat substrate and W2P2 had the largest diameter (5.9 mm) (see **Figure 3**). Transplants cultivated at W1P1, W2P1, and W2P3V3 exhibited a similar diameter and were also included in the same homogeneous group. The smallest shoot diameter was observed in plants from sites W1, W2, W1V1, and V (4.4-4.8 mm). Addition of peat to W1 and W2 substrates resulted in a statistically significant average increase in plant diameter of 24.6%. Mixing W2 compost with vermicompost in ratios of 50:50 or 75:25, and W2 compost with peat and vermicompost in a ratio of 50:25:25, resulted in an insignificant increase in shoot diameter by an average of 8.3%. However, altering the ratio in the W2P3V3 mixture to 75:10:15 resulted in a significant increase in diameter by 12.5% compared to W2.

The stem height:diameter ratio was determined by calculating the ratio of plant height to shoot diameter at the base and was statistically analysed (see **Figure 3A**). The plants grown in peat substrate had the lowest index (19.4), whereas those from homogeneous substrates W2, V, and W1 were significantly more stocky, with indices of 23.8, 25.5, and 26.3, respectively. The index from sites W1P1 and W1V1 fell into the same homogeneous group. On average, decreasing the percentage of peat in the substrates from 50%, to 25%, and 10% increased the index values to 23.4, 24.8, and 26.7. The stem height:diameter index also increased with decreasing the percentage of vermicompost in the substrates from 23 and 24 (50 and 25% share) to 26.7 (15% share), on average.

Upon statistical analysis, it was demonstrated that the addition of basaltmeal to the compost and peat mixture led to a 19.9% increase in the average plant diameter, which was equivalent to that of plants grown in pure peat substrate (as illustrated in **Figure 3B**). Conversely, the plants grown in substrates with added biochar exhibited a differential response. In this case, only the addition of a smaller dose of this component to the homogeneous compost substrate or to the compost and peat mixture resulted in a statistically significant increase in shoot diameter compared to W1 and W2. Only at the W1PC1 site (5.8 mm) did the plants reach the same diameter as those grown in pure peat (5.9 mm). Furthermore, a statistical comparison of the stockiness coefficients showed lower values for seedlings grown in media with added biochar, compared to plants from facilities where basaltmeal was utilized as an additive.

**Figure 4:**
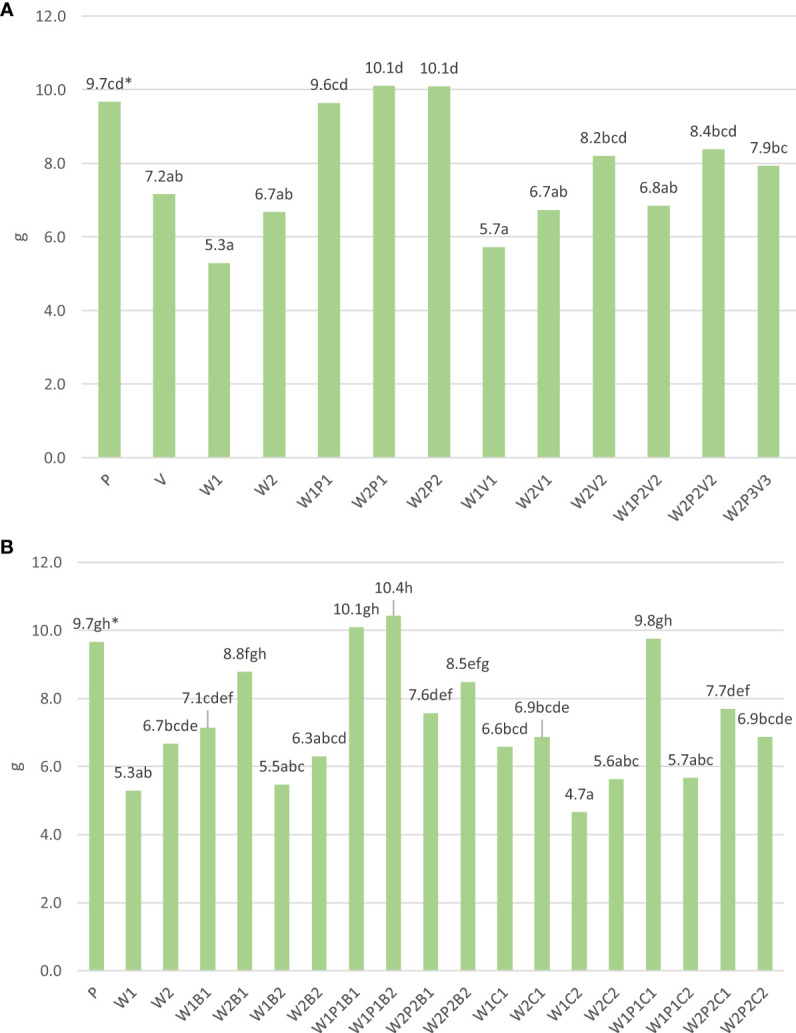
**(A)** The effect of horticulture media on one cucumber plant mass (g). *Means that include a common letter are not statistically significant. **(B)** The effect of horticulture media and additives on one cucumber plant mass (g). *Means include a lowercase letter are not statistically significant.

The study revealed that cucumber plants grown on substrates containing a combination of willow compost and peat in a ratio of 75% or 50% attained similar unit weights to those grown on pure peat substrate (**Figure 4A**). Plants from the W2P2V2 and W2P2 sites had statistically similar weights (10.1 g). In contrast, the addition of 50% vermicompost or pure willow compost to the substrate resulted in an average of 1.5 times lower plant weight.

Furthermore, the study found that the addition of basaltmeal at a rate of 5% (w/w) of substrate to W1 or W2 resulted in a 31.3% increase in plant weight (**Figure 4B**). When basaltmeal was added to compost-peat mixtures at a rate of 5% or 10% (w/w) of substrate, plant weight was almost twice as high (W1P1B1 and W1P1B2) or 1.2 times higher (W2P1B1 and W2P1B2) than the weight of plants grown on base compost substrates (W1 and W2). The addition of biochar to the substrates also had a beneficial effect on plant weight, resulting in an 84.9% and 14.9% increase in weight on W1PC1 and W2PC1, respectively, compared to plants grown on W1 and W2. The combined use of both additives had a favourable impact in the case of media containing peat as a component.

**Figure 5:**
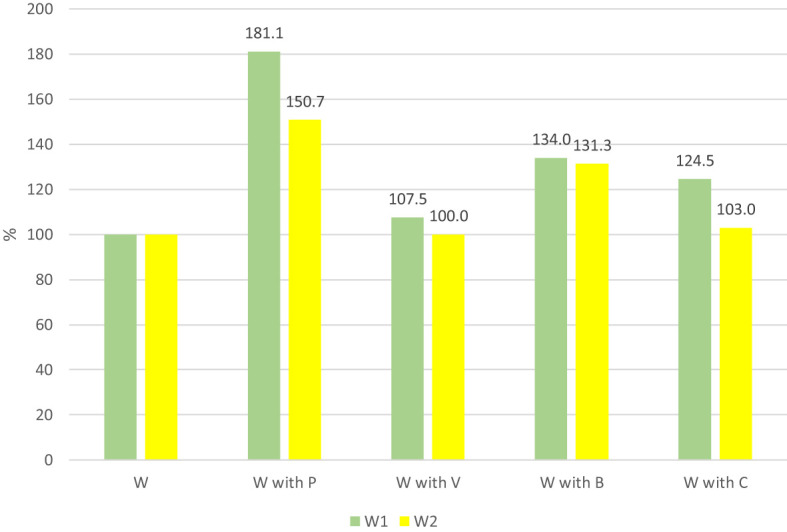
The effect of horticulture media additives on one plant mass changes (in %). The base value (100%) a cucumber plant mass on produced on compost media (W1 and W2) without media component and additives.

The findings show that cucumber transplants cultivated on W1 substrate with 50% of both initial composts exhibited a more pronounced response to the additives as compared to those grown in W2 substrate, as indicated in **Figure 5**. The results demonstrated that the inclusion of peat in the substrates led to the greatest increase in plant weight, by 81.1% and 50.7%, respectively. Moreover, the addition of basaltmeal to the substrates containing W1 and W2 resulted in a significant increase in plant unit weight of 34% and 31.3%, respectively, whereas the addition of biochar to the compost mixture with W1 led to a 24.5% increase in plant weight.

## Discussion

Among alternative material, willow biomass compost mixtures are a promising type of plant biomass that can significantly replace environmentally valuable peat. In UK conditions, wood-based raw materials are the primary alternative to peat (Holmes and Bain, 2023). In a previous study (Sowiński et al., 2022), willow composts were found to have no toxic effect on white mustard seed germination, and some aqueous extracts from compost-peat mixtures even showed a stimulating effect on white mustard seedlings. Composts made from cellulose-lignin biomass of willow (*Salix viminalis* L.) have shown suitability of for the production of horticulture media used in tomato and cucumber transplant as well as for lettuce cultivation (Adamczewska-Sowińska et al., 2021; Adamczewska-Sowińska et al., 2022; Bekier et al., 2022).

It was found that a substrate mixture (W2) composed of compost without additives and compost with added nitrogen and mycelium in a ratio of 75:25 was more effective than a mixture with an equal proportion of both composts (W1). In a study by Adamczewska-Sowińska et al. (2021), demonstrated that the C:N ratio in biomass is crucial not only during the willow composting process but also in seedling growth. The use of nitrogen-added homogeneous substrate made from willow compost resulted in plant deformation, which is typical of excessive nitrogen content. However, cucumber transplants did not exhibit adverse changes in the present study. The components and additives used to prepare the growing medium influenced the plant’s response, and the study demonstrated the differences in plant response.

Cucumber transplants were grown on various media, including W1P1, W2P1, W2P2, W1P1B1, W2P1B2, and W1P1C1, consisting of willow compost and 50 or 25% peat. Additionally, 5 or 10 g of basaltmeal per 100 g of substrate and 10 g of biochar were added to W1 and W2 compost mixtures. The results showed that the cucumber transplants growth was similar or even better than those grown in standard peat substrates. The quality of cucumber transplants declined when vermicompost (W1V1 and W2V2), higher amounts of basaltmeal (W1B2 and W2B2), or biochar (W1C2 and W2C2) were added to the W1 and W2 compost mixtures.

Several studies have suggested that up to 25% of peat in substrates can be replaced with different composts without negatively impacting tomato seedling quality. These findings are consistent with the results of the present study. However, the effects of vermicompost, basaltmeal, and biochar on cucumber growth were different in this study than in previous studies on different horticultural plant species. For example, Alvarez et al. (2017) recommend using 10 to 50% vermicompost as a substrate additive. Similarly, Jankauskiene et al. (2022) found that adding vermicompost to peat (up to 30%) had a beneficial effect on leaf number, cucumber plant height, and leaf area. In contrast, the present study found that the optimum proportion of vermicompost as an additive to willow composts was between 15-25%. Vermicomposting indirectly mitigates the effects of global warming and the greenhouse effect through its ability to sequester carbon media (Olle, 2019). The products obtained from vermicomposting processes depend on the feedstock used and their proportion in the mixtures. Final vermicompost product should be evaluated before utilisation considering its quality for different purposes such as organic fertilizer or substrate for seedlings production. Additionally, smaller amounts of basaltmeal (5 g per 100 g) and biochar (10 g per 100 g) as substrate additives were found to be more beneficial for cucumber growth. Basaltmeal as a rock dust is a mineral byproduct of mining, which can be used to improve the fertility of substrate, improve plant growth, enhance the activity of beneficial microflora and increase the quality of fruits and vegetables (Beerling et al., 2018). Application of basaltmeal stimulated plant growth and reduced the severity of bacterial wilt in greenhouse tomato production (Li and Dong, 2013). Alvarez et al. (2017) demonstrated that 12% biochar had no negative effect on petunia growth. Biochar improves physical properties of the substrate, including surface area, density, porosity, pore size, and water-holding capacity. Biochar surface area ranges from 100 to 800 m^2^/g, and porosity from 50 to 70% (Choi et al., 2019). In lettuce production (*Lactuca sativa* L.), replacing 10% of peat (in volume) by biochar, increased lettuce head mass by 184–270% compared to using peat substrate alone (Mendez et al., 2017).

A high-quality cucumber transplant is characterized by its health, developmental stage, optimal height-to-stem thickness ratio, short hypocotyl, thick green leaves and cotyledons, and well-developed roots (Yan et al., 2019; Li et al., 2013). In a recent study by Wang et al. (2021), cucumber seedlings achieved a height of 8.2-13.1 cm after 21 days of optimal cultivation, which is similar to the range of 9.7-15.5 cm observed in our experiment. However, the average plant weight of cucumber seedlings varied significantly between the two studies. In our study, it ranged from 5.3 to 10.4 g per plant, while in the study by Wang et al. (2021) it was much lower, ranging from 2.48 to 3.77 g. A more reliable indicator to assess plant growth dynamics during the initial phase is the height-to-diameter ratio (Haase, 2008; Díaz-Pérez and Camacho-Ferre, 2010; Adamczewska-Sowińska et al., 2022). In our study, the plants had a more uniform index ranging from 19.4 - for cucumber transplants produced in a peat medium (P) to 26.8 in the W1P1B1 medium.

## Conclusion

Growing media composed solely of a mixture of different willow composts, combined in varying proportions, did not provide equivalent growing conditions to that of the peat substrate during the initial growth period of cucumber plants. Conversely, the study demonstrated that the W1 and W2 mixtures can partially replace peat as one of the substrate components. Cucumber transplants with comparable or greater weight than those cultivated in peat substrate were obtained when the proportion of peat in the willow compost substrate ranged between 25-50%. Obtaining transplants with comparable characteristics to those grown in peat substrate, while utilizing up to 75% less of this valuable environmental material, holds significant importance for the development of this type of production. Furthermore, it should be emphasized that cucumber transplants cultivated on the optimal substrates containing willow compost were healthy, did not display any signs of disease, well develop, and maintained a height-to-stem thickness ratio over 25.

## Data availability statement

The original contributions presented in the study are included in the article/supplementary material. Further inquiries can be directed to the corresponding author.

## Author contributions

JS: Conceptualization, Data curation, Formal analysis, Funding acquisition, Investigation, Methodology, Project administration, Resources, Software, Supervision, Validation, Visualization, Writing – original draft, Writing – review & editing. KA-S: Conceptualization, Formal analysis, Investigation, Methodology, Validation, Writing – original draft, Writing – review & editing. EJ: Investigation, Methodology, Validation, Writing – review & editing. JB: Investigation, Methodology, Validation, Writing – review & editing.
